# “Sandwich Therapy”—Immunotherapy Plus Concurrent Chemoradiotherapy for Advanced Esophagogastric Junction Carcinoma: Report of Two Cases and Literature Review

**DOI:** 10.3389/fonc.2022.794153

**Published:** 2022-05-25

**Authors:** Lei Wu, Juan Liu, Long Liang, Mian Mao, Xiangpan Li, Tao Li, Jinyi Lang, Qifeng Wang

**Affiliations:** ^1^ Radiation Oncology Key Laboratory of Sichuan Province, Department of Radiation Oncology, Sichuan Cancer Hospital Institute, Sichuan Cancer Center, School of Medicine, University of Electronic Science and Technology of China, Chengdu, China; ^2^ Department of Oncology, Pidu District People’s Hospital, The 3rd Affiliated Hospital of Chengdu Medical College, Chengdu, China; ^3^ Department of Pharmacy, Sichuan Cancer Hospital and Institute, School of Medicine, University of Electronic Science and Technology of China, Chengdu, China; ^4^ Department of Oncology, Renmin Hospital of Wuhan University, Wuhan, China

**Keywords:** esophagogastric junction carcinoma, immunotherapy, chemotherapy, concurrent chemoradiotherapy, overall survival

## Abstract

**Introduction:**

Esophagogastric junction (EGJ) carcinomas develop in the transition zone between the esophagus and stomach. The incidence of EGJ carcinoma has steadily increased over the past few decades. Most patients are first diagnosed at an advanced stage, which renders them ineligible for surgery. Current methods for the treatment of advanced EGJ carcinoma include surgery, chemotherapy, local palliative therapy, and supportive care; however, none of these treatment methods has provided satisfactory therapeutic effects when used alone.

**Case Report:**

We report two cases of patients with EGJ carcinoma who were sequentially treated with immunotherapy plus induction chemotherapy, followed by immunotherapy plus concurrent chemoradiotherapy and maintenance immunotherapy. Both patients achieved extended overall survival times with good quality of life with this new therapeutic approach.

**Conclusion:**

Immunotherapy plus chemoradiotherapy may therefore be a reasonable option for treatment of selected EGJ carcinoma patients. However, well-designed trials for the acquisition of additional evidence are required to validate the findings in this study.

## Introduction

Esophagogastric junction (EGJ) carcinomas are malignant epithelial tumors of the gastrointestinal tract that occur at the transition zone between the esophagus and stomach. Over the past few decades, the incidence of EGJ carcinoma has increased globally (1). Current methods for the treatment of advanced EGJ carcinoma include surgery, chemotherapy, immunotherapy, local palliative radiotherapy and supportive care; however, none of these treatment methods has provided satisfactory therapeutic effects when used alone. A combination of multiple conventional treatment methods, such as surgery, radiotherapy, and chemotherapy has led to a decrease in the mortality rate for EGJ carcinoma; however, the five-year survival rate has remained low at 5–15% (2). In recent years, immunotherapy combined with chemotherapy has become the standard treatment recommended by the guidelines. In the phase III clinical study of pabolizumab or placebo combined with first-line chemotherapy for advanced esophageal cancer (keynote-590) (3), the median OS in the immune combined chemotherapy group was more than 12 months, and the efficacy exceeded the previous standard first-line chemotherapy. Another phase III clinical study (ESCORT-1) (4)showed that carilizumab combined with chemotherapy can significantly prolong the median survival (mOS, 15.3 months vs 12.0 months) and median progression free survival (mPFS, 6.9 months vs 5.6 months) of patients with advanced esophageal squamous cell carcinoma, and has good safety. In the subsequent CheckMate648, ORIENT-15, and JUPITER-06 (5–7) trials comparing a combination of immunotherapy and chemotherapy with chemotherapy alone, combination therapy was beneficial in terms of survival and increased the objective remission rate (ORR) compared to monotherapy (ORR increase from 27% to 45% in the chemotherapy-only group and from 45% to 72.1% in the combination therapy group). Although combination therapy exhibited higher efficacy and a good safety profile compared to monotherapy, the median survival of approximately 1 year remained unsatisfactory. Moreover, these trials did not provide data on interventions with local therapies, and it is unknown whether immunotherapy plus concurrent radiotherapy is safe and can further improve efficacy.

In some patients with advanced esophageal cancer who cannot undergo radical surgical resection, immunotherapy plus concurrent chemotherapy provides an opportunity for local treatment such as surgery or radiotherapy, but the timing of such treatment is unclear. In addition, some patients develop drug resistance after immunotherapy plus chemotherapy; therefore, it is unclear whether addition of radiotherapy provides a synergistic effect that reduces or delays development of drug resistance and improves patient prognosis. Herein, we report two cases of patients with advanced EGJ carcinoma in which remarkable benefits were achieved after treatment with immunotherapy plus chemoradiotherapy.

## Case Report

### Case 1

A 56-year-old man was admitted to our hospital in November 2019 with a one-month history of progressive dysphagia. Gastroscopy showed the presence of ulcerating neoplasms in the esophagus, beginning 36 cm caudal to the incisors and extending distally to involve the entire gastric cardia and areas of the proximal fundus and lesser curvature of the stomach. Gastric endoscopic ultrasonography revealed tumor extension into all five layers of the gastric walls of the cardia and fundus, serosal invasion of these structures, and enlargement of the adjacent lymph nodes. Histopathological examination of small biopsy specimens from the proximal esophageal and fundic portions of the tumor revealed the presence of atypical epithelial cells that were suspected to be cancerous. Immunohistochemical (IHC) stain results for the cells were as follows: P63 (+), CK8/18 (+), P40 (+), CDX-2 (±), CD56 (-), CgA (-), Syn (±), TTF-1 (-), Ki67 (approximately 90%), CK5/6 (+), CK20 (-). Programmed death-ligand 1 (PD-L1) expression was negative, and the combined positive score (CPS) was <10. A diagnosis of poorly differentiated squamous cell carcinoma (SCC) was considered based on the immunophenotyping results and cellular morphology. Contrast-enhanced magnetic resonance imaging (MRI) of the upper abdomen revealed the presence of: 1) multiple irregular soft tissue masses in the distal thoracic esophagus, the gastric cardia, and the lesser curvature near the gastric fundus and body, 2) poor serosal definition of these structures suggesting tumor invasion of the mesentery, 3) enlargement of left perihepatic and retroperitoneal lymph nodes, 4) blurred boundaries of the wall of the gastric lesser curvature, and 5) multiple organ metastases. The patient was diagnosed with poorly differentiated SCC of the EGJ with invasion of the gastric lesser curvature and left perihepatic lymph node metastases (cT4N3M0).

**Figure 1:**
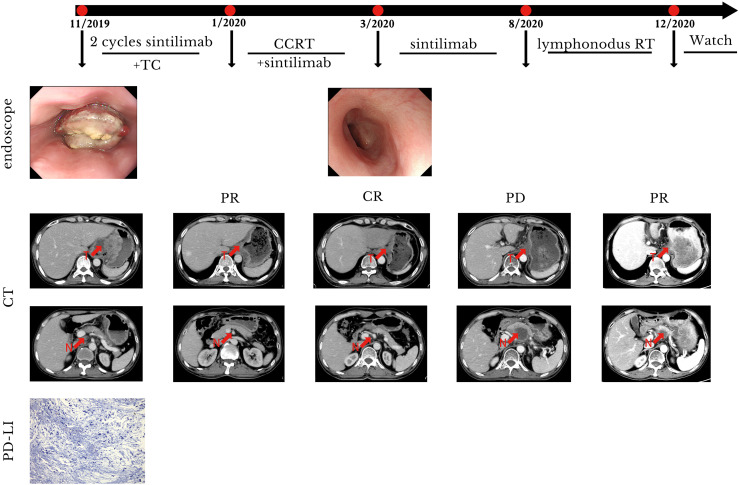
Case presentation of case 1. CCRT, concurrent radiotherapy; CR, complete remission; CT, computed tomography; N, lymphonodus; PR, partial remission; PD, progression disease; RT, radiotherapy; T, tumor; TC, paclitaxel/carboplatin.

**Figure 2:**
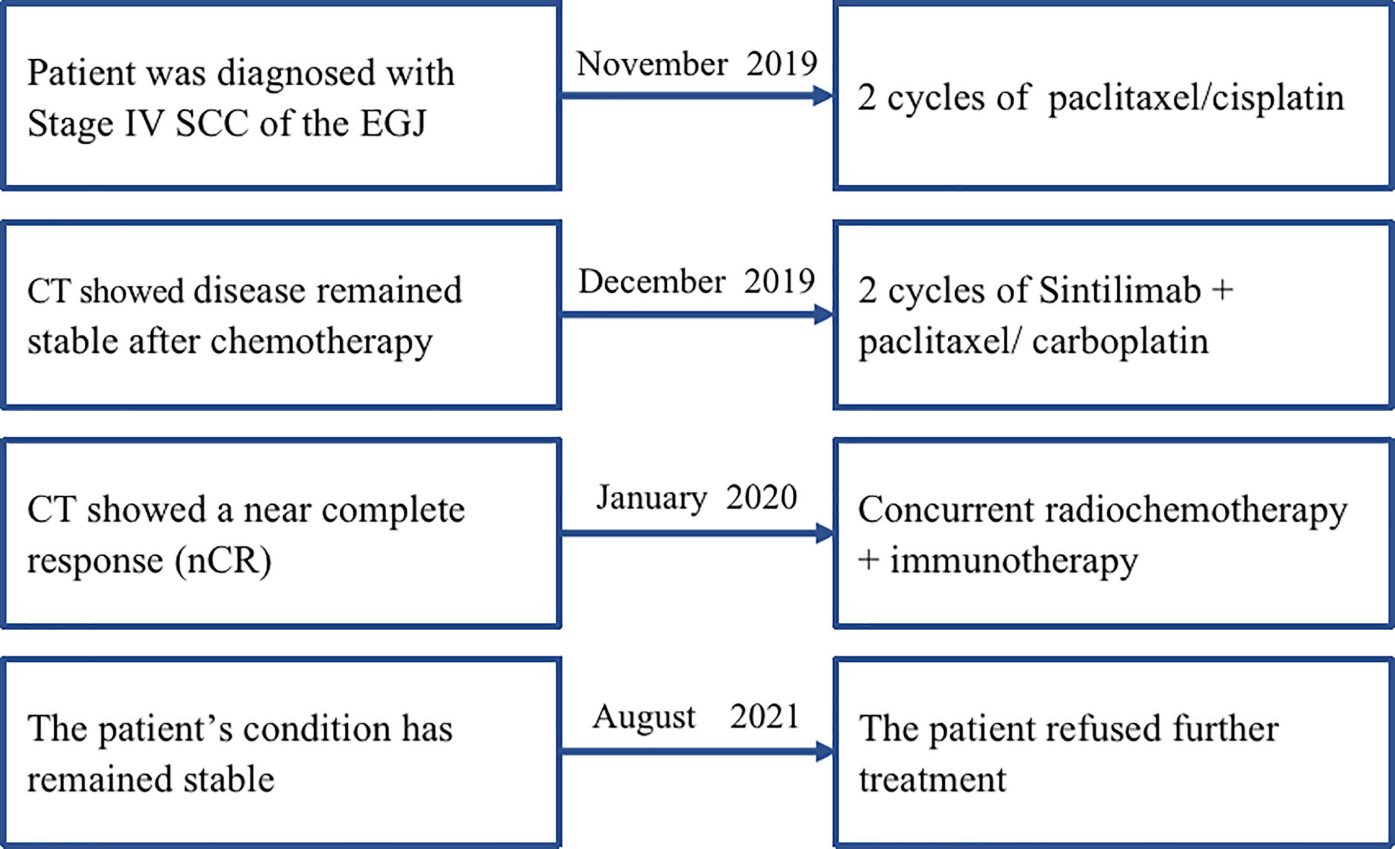
The timeline of diagnosis, interventions, and outcomes of case 1. SCC, squamous cell carcinoma; EGJ, esophagogastric junction; CT, computed tomography; cCCR, concurrent chemoradiotherapy.

Subsequently, two cycles of sintilimab (an anti-PD-1 monoclonal antibody) (day 1, 200 mg) and paclitaxel injections (day 1, 180 mg)/carboplatin (day 1, 300 mg) were administered from February to July 2019. Compared with the pre-treatment computed tomography (CT) scans, contrast-enhanced CT performed after two cycles of treatment demonstrated significant shrinkage of the primary tumors in the esophagus and complete disappearance of left perihepatic lymph node enlargement. Thus, a partial response (PR) was achieved for treatment of the tumor. Surgical treatment of the remaining tumor was recommended; however, the patient refused that option and requested subsequent radiotherapy. Treatment with radical concurrent chemoradiotherapy and immunotherapy was continued. Involved-field irradiation (IFI) was used for the delineation of target volumes to achieve targeted irradiation of the primary tumors and lymph node metastases. Considering that high irradiation doses might increase the risk of gastric perforation in the patient because of tumor invasion of the stomach, image-guided radiation therapy (IGRT) was administered at a dose of 1.8 Gy/fraction qd for 28 fractions (total dose: 50.4 Gy) to the gross tumor volume (GTV) at the target sites (primary esophageal tumors, enlarged lymph nodes). During radiotherapy, the combination immunotherapy-chemotherapy regimen previously described was concurrently administered for two cycles (four cycles in total). Sintilimab (200 mg, q3w) was administered after radiotherapy ended to serve as maintenance therapy. In August 2020, the tumor recurred in the abdominal lymph nodes and liver metastases were found. Consequently, radiotherapy was administered to the abdominal lymph nodes (45 Gy in 30 fractions over 3 weeks to the GTV of the nodes) and metastatic live lesions (50 Gy in 10 fractions over 2 weeks). Results of the latest imaging examination performed at our hospital in December 2020 indicated that the patient had achieved a complete response (CR) for the esophageal lesion and stable disease in the affected abdominal lymph nodes and metastatic liver lesions. To date, the patient is in a stable condition with no severe adverse reactions and an overall survival (OS) time that has exceeded 21 months (**Figure 1**). The timeline of diagnosis, interventions and outcomes was summarized as **Figure 2**.

### Case 2

**Figure 3:**
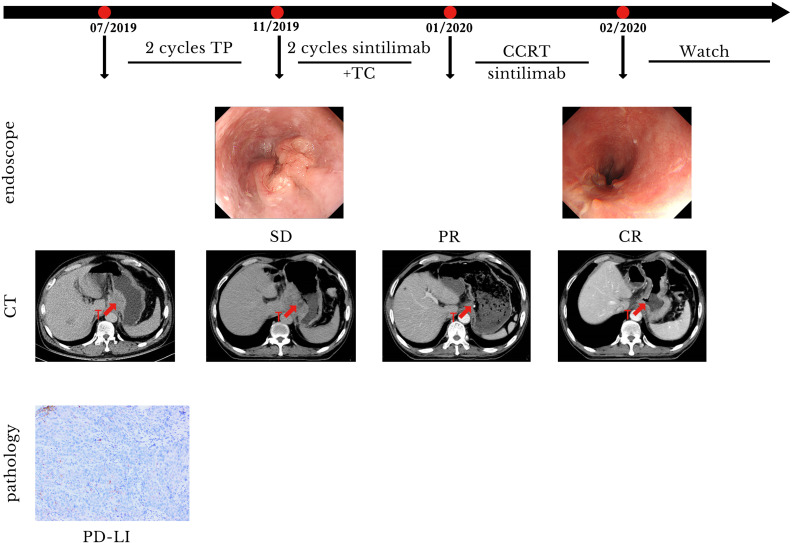
Case presentation of case 2. CCRT, concurrent radiotherapy; CR, complete remission; CT, computed tomography; PR, partial remission; SD, stable disease; T, tumor; TC, paclitaxel/carboplatin; TP, paclitaxel/cis-platinum.

**Figure 4:**
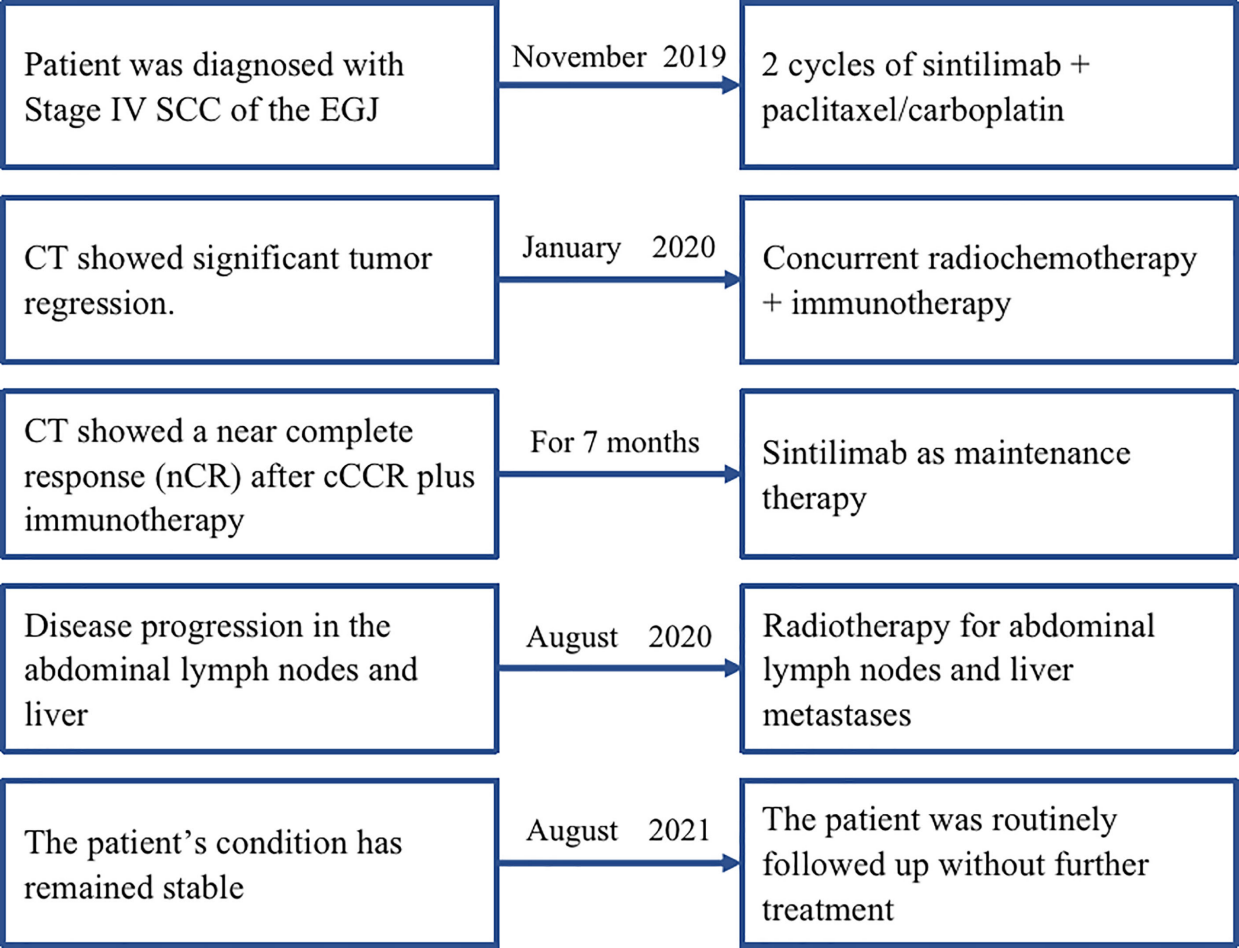
The timeline of diagnosis, interventions, and outcomes of case 2. SCC, squamous cell carcinoma; EGJ, esophagogastric junction; CT, computed tomography.

A 54-year-old man with a history of SCC of the distal esophagus was admitted to our hospital in November 2019, three weeks after a second cycle of chemotherapy. Gastroscopy showed the presence of lesions in the esophagus, extending from a site 30 cm distal to the incisors to the gastric cardia, which were suggestive for esophageal cancer. Histopathological examination of biopsy specimens revealed the presence of low-grade squamous intraepithelial lesions (at 20 cm distal to the incisors) and SCC (at 33 cm distal to the incisors). Contrast-enhanced CT identified thickenings of the gastric cardia wall at its junction with the esophagus and the wall of the lesser gastric curvature that suggested neoplasia. Contrast-enhanced MRI of the upper abdomen showed the presence of irregular mass-like thickened areas at the EGJ and in the wall of the lesser curvature near the fundus and gastric body, multiple masses attached to the pancreas, and multiple small and enlarged lymph nodes in the left perihepatic space, hepatic hilus, and retroperitoneal space. We evaluated PD-L1 expression in the specimens. The results showed that the tumor proportion score was <1% and the CPS was 5. The patient was diagnosed with SCC of the EGJ with invasion of the gastric lesser curvature and left perihepatic lymph node metastases (cT4N3M0). Prior to admission to our hospital, the patient had received two cycles of systemic chemotherapy with a paclitaxel + cisplatin regimen (unknown dose levels) at another hospital. Re-examination after chemotherapy indicated no significant tumor shrinkage had occurred. Therefore, we administered two cycles of sintilimab (day 1, 200 mg) plus paclitaxel injection (day 1, 180 mg)/carboplatin (day 1, 300 mg) from November to December 2019. Contrast-enhanced CT performed after two cycles of treatment revealed significant tumor shrinkage and the achievement of PR. On January 16, 2020, radiotherapy was commenced with the use of IGRT for targeted irradiation of tumors from the lower esophagus to the cardia and positive lymph nodes. Irradiation was administered qd in 25 fractions at a dose of 2.0 Gy/f for both the GTVs to the primary lesions in the esophagus and positive mediastinal lymph nodes. Similar to Case 1, two cycles of the immunotherapy plus chemotherapy regimen previously described above were concurrently administered to the patient during the course of radiotherapy treatment. Contrast-enhanced CT performed after radiotherapy demonstrated the achievement of a near complete response (nCR) in the esophageal tumors and lymph nodes.The patient refused maintenance immunotherapy and periodic re-examinations after completing radiotherapy. To date, the patient’s condition has remained stable; the quality of life is good with an absence of severe adverse reactions, and OS time has exceeded 21 months (**Figure 3**). The timeline of diagnosis, interventions and outcomes was summarized as **Figure 4**.

## Discussion

It has been previously reported that the use of chemotherapy alone as a first-line therapy for advanced esophageal carcinoma does not provide significant benefits (3). Therefore, the exploration of novel treatment methods for advanced esophageal and EGJ cancers is of utmost necessity. In the KEYNOTE-028 study conducted in 2014 (8), the anti-PD-1 antibody pembrolizumab was administered to patients with SCC or adenocarcinoma of the esophagus or EGJ for the first time. An overall response rate of 30% was achieved, and a good safety profile was demonstrated with no unexpected adverse reactions. The promising results of KEYNOTE-028 have led to many subsequent clinical studies on the treatment of advanced esophageal and EGJ cancers, including KEYNOTE-180, KEYNOTE-181, ATTRACTION-03 and ESCORT (9–12). These studies have demonstrated that the use of immunosuppressive agents as second-line or third-line therapy provides greater benefits to the prolongation of survival compared with chemotherapy alone. In the KEYNOTE-590 study (3), first-line chemotherapy plus pembrolizumab successfully improved the OS (12.4 months vs. 9.8 months) and progression-free survival of patients with advanced esophageal cancer compared with OS observed with first-line chemotherapy alone. In addition, the results of the study indicated that patients with lower PD-L1 expression also benefited from combination therapy.

In addition to standard chemotherapy, radiotherapy is an important palliative treatment option for patients with advanced esophageal carcinoma, and it can reduce the severity of dysphagia. Suzuki et al. (13) treated 31 patients who had stage IVB esophageal carcinoma with palliative radiotherapy at an external radiation dose of 30-60 Gy, which reduced the degree of dysphagia in 74% of patients. Jie Li et al. (14) conducted a retrospective study of 82 patients with heterochronic, oligometastatic esophageal cancer. The authors divided the patients into radiotherapy and non-radiotherapy groups, which exhibited median OS values of 14 (95% confidence interval [CI], 11.0–17.0) and 7 (95% CI, 4.5–9.5) months, respectively (P = 0.016). Cox multivariate regression analysis revealed that the treatment modality (radiotherapy vs. non-radiotherapy) was an independent prognostic factor for oligometastatic esophageal carcinoma (hazard ratio: 1.8). No toxic side effects greater than grade 3 were observed in either group. These studies suggest that radiotherapy may prolong survival in patients with advanced esophageal cancer, but most previous clinical studies of advanced esophageal carcinoma have focused on the combination of immunotherapy and chemotherapy, with the role of radiotherapy in immunotherapy-based combination therapy remaining unclear. Radiotherapy combined with immunotherapy enables the effective stimulation of the systemic anti-tumor immune response, which enhances the effector functions of the T-cell response and provides killing effects on tumors in distant locations and non-irradiated sites. Therefore, the combination of radiotherapy and immunotherapy produces synergistic anti-tumor effects, which may potentially increase the cure rate of tumors (15). However, given the current lack of standard treatment methods for radiotherapy combined with immunotherapy and chemotherapy, further clinical research is required for the elucidation of target volume delineation, dose fractionation schemes, total radiation dose, and timing of incorporation of radiotherapy into the treatment regimen.

The present case reports describe the novel approach of two cycles of induction chemotherapy plus immunotherapy for the treatment of advanced EGJ carcinoma. Induction therapy reduces tumor volume and tumor load by first alleviating symptoms of dysphagia and consequently improving nutrition and general physical health. The adoption of immunotherapy prior to radiotherapy not only blocks primary tumor growth and micrometastatic disease, but also utilizes the high levels of endogenous tumor-associated antigens present in the primary tumors to promote T-cell activation, thereby producing wider-ranging anti-tumor immune responses (16). Furthermore, tumor shrinkage creates favorable conditions for subsequent concurrent chemoradiotherapy, which include considerably reduced target volumes for radiotherapy, alleviation of toxic side effects caused by radiotherapy, lower risk of bleeding and perforation in tumors and the gastrointestinal tract, and better protection of normal surrounding tissue. In the case studies, the total radiation dose was also controlled within 50 Gy, and IFI was adopted for target volume delineation to further reduce the extent of irradiation to minimize the potential toxic effects caused by the introduction of radiotherapy into the treatment regimens. The use of radiotherapy may also provide an opportunity for salvage surgical treatment after tumor progression in some patients with advanced esophageal carcinoma. Both patients described in this report achieved an OS time of more than 21 months, which far exceeds the median survival time of 12.4 months reported in a study that included 749 patients with advanced esophageal cancer (3). Although such effects may be sporadic, our results still indicate that immunotherapy plus concurrent chemoradiotherapy is a potential treatment option for patients with advanced EGJ carcinoma. Concurrent immunotherapy should be continued during chemoradiotherapy, but this triple combination therapy increases the intensity of treatment and raises safety concerns. However, several clinical trials (17–19) have used a combination of concurrent radiotherapy and PD-1 inhibitors for the treatment of locally advanced esophageal cancer, suggesting that triple combination therapy is a viable treatment modality for some patients with esophageal cancer. In addition, both patients were negative for PD-L1 expression, suggesting that some patients with low PD-L1 expression might benefit from the combination with immunotherapy.

In conclusion, the “sandwich therapy” approach of immunotherapy plus chemotherapy, sequentially followed by concurrent chemoradiotherapy and maintenance immunotherapy, may be a reasonable treatment option for patients with advanced EGJ carcinoma. To validate the findings of this case report, the acquisition of better scientific evidence through well-designed trials is required. Presently, we have registered a clinical trial to investigate the efficacy and safety of toripalimab in combination with standard chemotherapy followed by concurrent chemoradiotherapy as treatment for treatment-naïve stage IV esophageal squamous cell carcinoma (Registration No.: ChiCTR2100046715). The results of this clinical trial will aid in the elucidation of the role and effects of radiotherapy in immunotherapy-based combination therapy for esophageal carcinoma and exploration of better combination therapy approaches.

## Data Availability Statement

The original contributions presented in the study are included in the article/supplementary material. Further inquiries can be directed to the corresponding authors.

## Ethics Statement

Written informed consent was obtained from the individual(s) for the publication of any potentially identifiable images or data included in this article.

## Author Contributions

LL and MM provided patient information. LW and JL collected the data. LW and QW were responsible for study conception and design and acquiring financial support. TL, JYL, and XL critically revised the manuscript for intellectual content. All authors contributed to the article and approved the submitted version.

## Funding

This study was supported by grants from Sichuan Science and Technology Department Key Research and Development Project Fund [2019YFS0378].The funding sources played no role in the study’s design, data analysis, or decision to publish the findings.

## Conflict of Interest

The authors declare that the research was conducted in the absence of any commercial or financial relationships that could be construed as a potential conflict of interest.

## Publisher’s Note

All claims expressed in this article are solely those of the authors and do not necessarily represent those of their affiliated organizations, or those of the publisher, the editors and the reviewers. Any product that may be evaluated in this article, or claim that may be made by its manufacturer, is not guaranteed or endorsed by the publisher.
